# 641. Outcomes Among Patients Referred with Concern for SJS/TEN: One Burn Center's 10 Year Experience

**DOI:** 10.1093/jbcr/irag033.478

**Published:** 2026-04-06

**Authors:** Raquel Arias-Camison, Bashar M Alsamman, Derek Bell

**Affiliations:** University of Rochester Medical Center, Rochester, NY; University of Rochester Medical Center, Rochester, NY; University of Rochester Medical Center, Rochester, NY

## Abstract

**Introduction:**

Stevens Johnson Syndrome and Toxic Epidermal Necrolysis (SJS/TEN) require urgent multidisciplinary management, however triage criteria for referral is variable. We characterized our burn center’s experience with patients referred with concern for SJS/TEN compared to patients with confirmed diagnoses and explore factors associated with each group.

**Methods:**

Patients referred from 2015-2025 were retrieved from the burn center registry. Chart review was performed to confirm initial referral with concern for SJS/TEN, final clinical diagnosis, pathology, and mortality with additional data retrieved from the burn center registry. Patients who were not admitted to the hospital or whose initial referral did not indicate concern for SJS/TEN were excluded from the cohort. Descriptive statistics were used.

**Results:**

Seventy-four patients were referred to the burn center with concern for SJS/TEN from 2015-2025; of these, 22 (29.7%) were ultimately diagnosed with SJS/TEN. Demographics were similar among both groups. The total affected body surface area was also similar across both groups and there was no difference in the source of referrals between the patient groups. Of patients who were referred and ultimately determined not to have SJS/TEN, the diagnoses were mostly other types of drug reactions (34.6%), rashes of infectious etiology (25%), or dermatitis (15.4%). Biopsy with dermatopathology was more commonly used to confirm these diagnoses than SJS/TEN (46.2% vs 22.7% in the SJS/TEN group, *p*=.06). Death was rare in both groups (*p*=.83).

**Conclusions:**

Our findings affirm existing literature that among patients referred to a burn center with concern for SJS/TEN a minority truly have the condition and the diagnosis is not associated with TBSA. Our study adds that tissue biopsy may be most useful for ruling out cases of SJS/TEN rather than confirming it. Additionally, our mortality rate among SJS/TEN patients was lower than reported in some literature, warranting further investigation into factors other than appropriate triage and biopsy-confirmed diagnosis in driving outcomes among this patient population.

**Applicability of Research to Practice:**

This study highlights the need for more standardized triage and diagnosis of SJS/TEN for referral to burn centers.

**Funding for the study:**

N/A.

